# Insulin Glargine Combined with Oral Antidiabetic Drugs for Asians with Type 2 Diabetes Mellitus: A Pooled Analysis to Identify Predictors of Dose and Treatment Response

**DOI:** 10.1007/s13300-018-0381-9

**Published:** 2018-03-09

**Authors:** Tianwei Gu, Ting Hong, Pengzi Zhang, Sunyinyan Tang, Yan Bi, Hai Lu, Lichuang Men, Dongwei Ma, Dalong Zhu

**Affiliations:** 1 Department of Endocrinology, Drum Tower Hospital Affiliated to Nanjing University Medical School, Nanjing, China; 2 Sanofi Investment Co., Ltd., Shanghai, China

**Keywords:** Asia, Basal insulin, China, Dose titration, Insulin glargine 100 U/mL, Nomogram, Pooled analysis, Type 2 diabetes mellitus

## Abstract

**Introduction:**

In Asia, patients with type 2 diabetes mellitus (T2DM) often have suboptimal glycemic control for many years prior to initiating basal insulin. Active titration of basal insulin is also required to improve glycemic outcomes. This pooled analysis was conducted to determine the impact of patient baseline covariates on the required dose of basal insulin and treatment response, for the improved management of Asian patients with T2DM.

**Methods:**

Data on insulin-naïve Asian patients with T2DM who initiated and fully titrated insulin glargine 100 U/mL (Gla-100) for ≥ 20 weeks were pooled from seven randomized, controlled, treat-to-target trials. Covariance and multivariate linear/logistic regression analyses were applied to determine the impact of the baseline covariates on Gla-100 dose (primary outcome) and treatment response (secondary outcomes) at week 24 for patients from Asia (*N* = 724) and from China alone (*n* = 249). Based on the multivariate analysis for the primary outcome in the Asian population, a nomogram was developed.

**Results:**

The dose of Gla-100 at week 24 was negatively correlated with age and positively correlated with body mass index (BMI) and fasting plasma glucose (FPG) at baseline in both Asian and Chinese populations. In both populations, higher baseline glycated hemoglobin (HbA_1c_) was associated with a lower reduction in HbA_1c_ from baseline, higher HbA_1c_ at week 24, and a lower chance of achieving HbA_1c_ < 7% at week 24. The constructed nomogram enables calculation of the likely dose of Gla-100 required by Asian patients with T2DM to achieve HbA_1c_ < 7% at week 24.

**Conclusions:**

Higher doses of Gla-100 are likely to be required in younger patients or patients with higher baseline BMI or FPG. The nomogram developed in this study can aid clinicians to titrate the dose of Gla-100 appropriately. Evidence in this pooled analysis also indicates that initiating basal insulin at a lower HbA_1c_ can lead to greater glycemic control.

**Funding:**

Sanofi China Investment Company.

**Electronic supplementary material:**

The online version of this article (10.1007/s13300-018-0381-9) contains supplementary material, which is available to authorized users.

## Introduction

Type 2 diabetes mellitus (T2DM) is a progressive disease often requiring add-on therapy, as many patients remain at suboptimal glycemic control on oral antidiabetic drugs (OADs) alone [1, 2]. The addition of basal insulin is recommended by guidelines as one of the initial treatment intensification options in these patients [3–6]. Despite recommendations for timely initiation, data indicate that Asian patients with T2DM have suboptimal glycemic control for approximately 6–9 years, with average glycated hemoglobin (HbA_1c_) levels of 9.2–10.5% at the point of basal insulin initiation [7–9]. In Asian patients, delays in basal insulin initiation are associated with a lower reduction in HbA_1c_, as well as a lower proportion of patients being able to achieve HbA_1c_ < 7% [7].

In addition to the issues surrounding timely initiation of basal insulin in Asia, there are several challenges pertaining to the titration of basal insulin. In clinical practice, Asian patients with T2DM who have a lower body mass index (BMI) than their Caucasian counterparts [10–12] are often perceived to be at an increased risk of hypoglycemia, which leads to conservative treatment goals and a cautious approach to dose titration by physicians [9, 13]. Large, real-world studies conducted in Asia, such as The First Basal Insulin Evaluation (FINE) Asia registry study and the Observational Registry of Basal Insulin Treatment (ORBIT) study, have hypothesized that further active titration of basal insulin can potentially increase the proportion of patients with T2DM achieving adequate glycemic control and improve glycemic outcomes [9, 14].

There is a growing body of evidence describing the importance of individualizing the management of T2DM based on ethnicity [15–17]. Therefore, when titrating the dose of basal insulin, it is important to consider the ethnic and genetic differences between Asians and Caucasians [18, 19], which may lead to different insulin requirements in both of these populations [20]. Understanding the specific insulin needs of Asian populations requires greater understanding of the patient-level factors that impact their required dose of basal insulin and treatment response.

The current study is a pooled analysis of Asian patients included in treat-to-target (TTT) randomized controlled trials (RCTs) of insulin glargine 100 U/mL (Gla-100). It was designed to investigate the relationship between patient baseline covariates, basal insulin dose, and treatment outcomes in insulin-naïve Asian patients with T2DM who have suboptimal glycemic control with OADs. To further explore the relationship between patient characteristics and outcomes, separate analyses were performed for the main population of patients from Asia and for a subpopulation of patients from China. The aim was not to compare both populations, but rather to lay the foundation for future analyses specific to the Chinese population and provide information to guide local clinical practice.

## Methods

### Study Design and Population

This was a pooled analysis of Asian patients included in seven TTT RCTs [1, 21–27]. Details of the seven RCTs, including their respective titration algorithms, are provided in Table 1. Trials were identified on the basis of a search of the study sponsor’s internal database for all TTT RCTs that it has conducted involving treatment with Gla-100 for at least 24 weeks in patients with T2DM uncontrolled with OADs. A total of 14 trials were identified; however, seven were excluded on the basis of the small number of patients in each trial fulfilling the inclusion criteria. Inclusion criteria were any insulin-naïve Asian patient in the full analysis set (FAS) population of the individual trials aged > 18 years with T2DM previously uncontrolled with OADs, with HbA_1c_ ≥ 7.0% and ≤ 12.0%, who had initiated and fully titrated Gla-100 according to a TTT algorithm for at least 20 weeks during the course of the individual RCTs. Patients were excluded in the individual trials if they had diabetes other than T2DM, acute diabetic complications (including unexplained severe hypoglycemia in the last 6 months), clinically significant acute major organ or systemic disease, or if they were pregnant or lactating. As this was a pooled analysis of pre-existing data, no ethical approval was required to conduct the study. This article is based on previously conducted studies and does not involve any new studies of human or animal subjects performed by any of the authors.

**Table 1:** Summary of the treat-to-target trials included in the pooled analysis

Study	Phase	Treatment	Treatment duration (weeks)	Glycemic target (mmol/L)	Titration schedule for Gla-100
ATLAS [22, 24]	IV	Gla-100 physician-led titration vs. Gla-100 patient-led titration	24	FBG < 6.1	Starting dose: 10 U/day^a^ Titration: conducted by physicians at each study visit or by patients twice per week on the basis of the intermediate value of the last three consecutive FBG values Algorithm FBG ≤ 3.1 mmol/L: dose decreased at physician’s discretion and upon physician’s clinical judgment FBG ≤ 3.9 mmol/L or symptomatic hypoglycemia: dose decreased by 2 U 6.1 mmol/L ≥ FBG > 3.9 mmol/L: no change in the dose 8.9 mmol/L ≥ FBG > 6.1 mmol/L: dose increased by 2 U FBG > 8.9 mmol/L: dose increased by 4 U
GALAPAGOS [21]	IV	Gla-100 vs. premixed insulin	24	BG 4.4–5.5	Starting dose: 0.2 U/kg or 12 U Titration: conducted by patients every 3 days and based on the median of the last three SMBG values^b^ Algorithm BG < 4.4 mmol/L or symptomatic hypoglycemia: dose decreased by 2 U 5.5 mmol/L ≥ BG ≥ 4.4 mmol/L: no change in dose 7.8 mmol/L ≥ BG ≥ 5.6 mmol/L: dose increased by 2 U BG > 7.8 mmol/L: dose increased by 4 U
L2T3 [26]	IV	Gla-100 vs. insulin detemir	24	SMFPG < 5.6	Starting dose: 0.2 U/kg Algorithm: doses increased by patients every 2 days by 2 U until target SMFPG was reached
Study 4001 [23]	IV	Gla-100 vs. NPH insulin	24	FBG ≤ 5.6	Starting dose: according to Holman and Turner’s formula Titration: conducted at every physician visit, based on whether patient’s FBG was greater than the values below for at least 1–2 consecutive days prior to the physician visit Algorithm FBG > 5.6 mmol/L: dose increased by 2 U FBG > 6.7 mmol/L: dose increased by 4 U FBG > 7.8 mmol/L: dose increased by 6 U FBG > 8.9 mmol/L: dose increased by 8 U
INSIGHT [25]	IV	Gla-100 vs. OAD therapy	24	FPG ≤ 5.5	Starting dose: 10 U Algorithm: doses increased by the patient by 1 U every day until target FPG was reached
INITIATE [27]	IV	Gla-100 group education vs. Gla-100 individual education	24	FPG 4.0–5.5	Starting dose: 10 U/day Algorithm: doses increased by patients by 2–4 U when FPG exceeded 5.5 mmol/L for 3 consecutive days. If FPG < 4.0 mmol/L and symptomatic hypoglycemia occurred without an identifiable reason, doses were decreased by 2 U/day
The treat-to-target trial [1]	III	Gla-100 vs. NPH insulin	24	FPG ≤ 5.5	Starting dose: 10 U/day Titration: conducted weekly during study visits or through telephone calls, based on the mean of SMFPG values from the preceding 2 days^c^ Algorithm FPG 5.6–6.7 mmol/L: dose increased by 2 U FPG 6.7–7.8 mmol/L: dose increased by 4 U FPG 7.8–10.0 mmol/L: dose increased by 6 U FPG ≥ 10 mmol/L: dose increased by 8 U

*BG* blood glucose, *FBG* fasting blood glucose, *FPG* fasting plasma glucose, *Gla-100* insulin glargine 100 U/mL, *NPH* neutral protamine Hagedorn, *OAD* oral antidiabetic drug, *SMBG* self-monitored BG, *SMFPG* self-monitored FPG

^a^The starting dose was modified where local practices required otherwise: 8–10 U/day for India and 4 U/day for subjects in Japan

^b^Patients used the median of the last three SMBG values, with the exception that the lowest value was used if it was < 4.4 mmol/L

^c^Exceptions to this algorithm included no increase in dosage if FPG < 4 mmol/L was documented at any time in the preceding week, and in addition to no increase, small dose decreases (2–4 U/day per adjustment) if severe hypoglycemia (requiring assistance) or FPG < 3.1 mmol/L were documented in the preceding week

### Study Outcomes

The primary outcome of this pooled analysis was identification of the baseline predictors of the dose of Gla-100 at week 24 in both the Asian population and in the subpopulation of patients from China. Based on the identified baseline predictors in the Asian population, a nomogram was developed for numerical determination of the dose of Gla-100 likely to be required to achieve target glycemic control (HbA_1c_ < 7%) at week 24. Secondary outcomes were identification of the baseline predictors for treatment response and the dose of Gla-100 in units per kilogram per day at week 24 in both study populations. The different measures of treatment response were HbA_1c_ value at week 24, achievement of target glycemic control (HbA_1c_ < 7%) at week 24, HbA_1c_ response (calculated as HbA_1c_ at week 24 − baseline HbA_1c_), and fasting plasma glucose (FPG) level at week 24.

### Statistical and Analytical Procedures

Eligible patients who were in the FAS population of the seven individual RCTs comprised the analysis population of this study. The baseline covariates explored were age, sex, weight, BMI, duration of diabetes, FPG, postprandial plasma glucose (PPG), HbA_1c_, and number of OADs prescribed. Correlation analyses were performed among the baseline covariates for the primary and secondary outcomes using analysis of covariance (ANCOVA). Spearman correlation coefficients and their associated *P* values were computed for each pairwise correlation.

Following the covariance analyses, multivariate analyses were conducted. Forward selection of the baseline covariates from the covariance to the multivariate models was performed on the basis of an entry selection criterion of *α* = 0.2. A multivariate, generalized linear regression model was run with all the baseline covariates that satisfied the forward model selection criterion. Parameter estimates and their associated 95% confidence intervals (CI) and *P* values were calculated for the selected baseline covariates included in the multivariate model.

For achievement of target glycemic control at week 24, a logistic regression model was used for the covariance analysis, and a multivariate logistic regression model was run with all the baseline covariates that satisfied the entry selection criterion, in accordance with the other analyses.

The nomogram was constructed on the basis of results of the multivariate analysis of the primary outcome in the FAS Asian population. In developing the nomogram, the linear predictor method was used to assign points to characteristics and predictions from the multivariate model to map cumulative point totals. The nomogram is a representation of the results of the multivariate analysis as a whole and includes all of the patient baseline covariates within the analysis, regardless of whether they were significant or not.

All analyses were conducted separately in the FAS Asian population and subpopulation of patients from China. Data were analyzed using SAS 9.2 (Cary, NC, USA) or a later version, and the nomogram was plotted using SAS 9.4 (Cary, NC, USA). Variables with *P* < 0.05 in the covariance or multivariate analyses were considered as significant predictors of the primary and secondary outcomes.

## Results

### Patients

#### Participant Selection

A total of 724 Asian patients from the seven TTT RCTs satisfied the study eligibility criteria and were included in this pooled analysis. The majority of patients (including the subgroup of 249 patients from China) were from the ATLAS [22, 24] and GALAPAGOS [21] studies. Details of the studies are provided in Table 1 and patient disposition is shown in Table S1 in the supplementary material.

#### Baseline Characteristics

The baseline characteristics of included patients are summarized in Table 2. Overall, 34.4% of patients were from China and the remainder were from other Asian countries. In the Asian population, mean age was 55.9 ± 8.79 years, 56.9% were male, and 82.7% were receiving two OADs at the time of the study. In the Chinese subpopulation, mean age was 57.7 ± 8.21 years, 52.6% were male, and 83.5% were receiving two OADs.

**Table 2:** Baseline characteristics of the study population

Category	All patients (*N* = 724)	Chinese patients (*n* = 249)
Age, years		
*N*	723	248
Mean (SD)	55.9 (8.79)	57.7 (8.21)
Sex, *n* (%)		
Female	311 (43.0)	117 (47.0)
Male	412 (56.9)	131 (52.6)
Missing	1 (0.1)	1 (0.4)
Race, *n* (%)		
Asian/oriental	724 (100.0)	249 (100.0)
Country, *n* (%)		
China	249 (34.4)	249 (100.0)
Others	475 (65.6)	0
Weight, kg		
*N*	724	249
Mean (SD)	70.2 (12.41)	69.4 (11.03)
BMI, kg/m^2^		
*N*	724	249
Mean (SD)	26.4 (4.02)	25.5 (3.36)
Duration of diabetes, years^a^		
*N*	711	249
Mean (SD)	9.7 (6.28)	9.6 (5.78)
FPG, mmol/L		
*N*	718	249
Mean (SD)	9.0 (2.30)	8.9 (2.08)
PPG, mmol/L		
*N*	691	245
Mean (SD)	12.8 (3.67)	12.4 (2.91)
HbA_1c_, %		
*N*	724	249
Mean (SD)	8.7 (1.03)	8.5 (1.05)
Number of OADs		
0	4 (0.6%)	0
1	66 (9.1%)	41 (16.5%)
2	599 (82.7%)	208 (83.5%)
3	54 (7.5%)	0
4	1 (0.1%)	0

*BMI* body mass index, *FPG* fasting plasma glucose, *HbA*_*1c*_ glycated hemoglobin, *OADs* oral antidiabetic drugs, *PPG* postprandial glucose, *SD* standard deviation

^a^Duration of diabetes = informed consent form signed date − diabetes start date. If the diabetes start date was partial or missing, 6 was used to impute the month and 15 was used to impute the day

#### Patient Data at Week 24

Endpoint patient data at week 24 are summarized in Table 3. In the Asian population, the final dose of Gla-100 was 25.7 ± 17.31 U (0.4 ± 0.23 U/kg/day), HbA_1c_ response was − 1.3 ± 1.10%, HbA_1c_ value was 7.5 ± 0.96%, and FPG was 6.1 ± 1.52 mmol/L. Amongst the Asian population, 31.9% achieved target glycemic control. In the Chinese subpopulation, the final dose of Gla-100 was 22.4 ± 11.20 U (0.3 ± 0.15 U/kg/day), HbA_1c_ response was − 1.3 ± 1.07%, HbA_1c_ value was 7.2 ± 0.82%, and FPG was 6.0 ± 1.23 mmol/L. Amongst the Chinese subpopulation, 41.4% achieved target glycemic control.

**Table 3:** Patient data at week 24

Category	All patients (*N* = 724)	Chinese patients (*n* = 249)
FPG, mmol/L		
Mean (SD)	6.1 (1.52)	6.0 (1.23)
Gla-100 dose, U		
Mean (SD)	25.7 (17.31)	22.4 (11.20)
Gla-100 dose, U/kg/day		
Mean (SD)	0.4 (0.23)	0.3 (0.15)
HbA_1c_, %		
Mean (SD)	7.5 (0.96)	7.2 (0.82)
HbA_1c_ response, %		
Mean (SD)	− 1.3 (1.10)	− 1.3 (1.07)
Achieving target glycemic control		
HbA_1c_ < 7%, *n* (%)	231 (31.9%)	103 (41.4%)
HbA_1c_ ≥ 7%, *n* (%)	493 (68.1%)	146 (58.6%)

*FPG* fasting plasma glucose, *Gla-100* insulin glargine 100 U/mL, *HbA*_*1c*_ glycated hemoglobin, *SD* standard deviation

### Primary Outcome

**Fig. 1:**
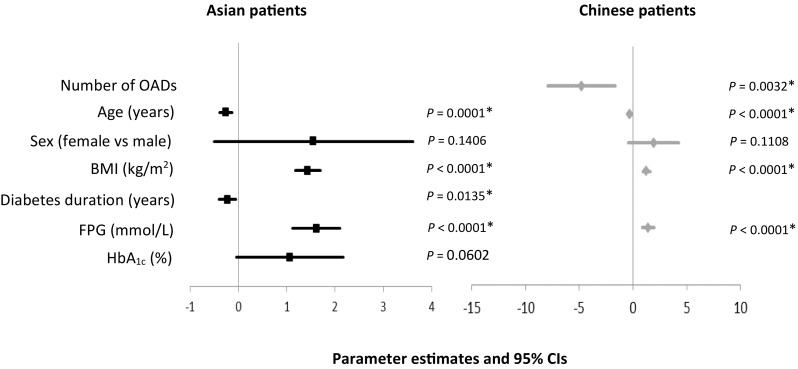
Parameter estimates and 95% confidence intervals for baseline predictors of Gla-100 dose at week 24 following multivariate regression analyses. Results are summarized for covariates included in the final model for each population. For the overall Asian population, the number of OADs prescribed at baseline did not satisfy the forward model entry selection criterion (from univariate to multivariate analysis) and hence was not included in the multivariate analysis. Similarly, for the Chinese patients, duration of diabetes and HbA_1c_ at baseline both failed to satisfy the entry criterion from univariate to multivariate analysis. Baseline covariates not included in the multivariate analyses for both populations have been left blank. *BMI* body mass index, *CI* confidence interval, * FPG* fasting plasma glucose, *Gla-100* insulin glargine 100 U/mL, *HbA*_*1c*_ glycated hemoglobin, *OADs* oral antidiabetic drugs. *Statistically significant

Following multivariate regression analysis in the Asian population, BMI, FPG, duration of diabetes, and age at baseline were significantly associated with the dose of Gla-100 at week 24 (Fig. 1 and Table S2). Increases in BMI by 1 kg/m^2^ and FPG by 1 mmol/L at baseline were associated with an increase in Gla-100 dose at week 24 by 1.44 U (*P* < 0.0001) and 1.62 U (*P* < 0.0001), respectively. An increase in the duration of diabetes by 1 year prior to the initiation of insulin was associated with a decrease in the dose of Gla-100 at week 24 by 0.23 U (*P* = 0.0135). Similarly, an increase in age by 1 year at baseline was associated with a reduction in the dose of Gla-100 by 0.26 U (*P* = 0.0001) at week 24.

**Fig. 2:**
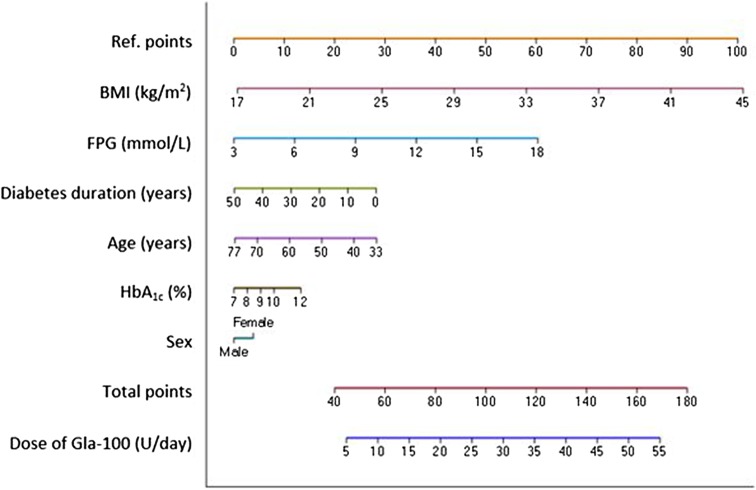
Nomogram to predict the dose of Gla-100 likely to be required to achieve target glycemic control at week 24. Note: choose the appropriate value of each of the baseline covariates, intercept perpendicularly the top horizontal line (Ref. points) and read the number. The sum of the ref. points, plotted on the “total points” line, corresponds to the prediction of the dose requirement for a patient at 24 weeks of treatment with Gla-100. For example, a 60-year-old female patient who has had T2DM for a duration of 10 years (ref. points with BMI 25 kg/m^2^, FPG 12 mmol/L, and HbA_1c_ 10%, approximately 113 total points) will likely require an insulin dose of 32 U/day at week 24 to achieve target glycemic control. *BMI* body mass index, *FPG* fasting plasma glucose, *Gla-100* insulin glargine 100 U/mL, *HbA*_*1c*_ glycated hemoglobin

Figure 2 presents the nomogram with which to calculate the likely required dose of Gla-100 to achieve target glycemic control at week 24, in Asian patients with T2DM. The dose of Gla-100 is calculated using all of the baseline covariates that were included in the multivariate analysis. The nomogram demonstrates that higher baseline BMI, FPG, and HbA_1c_, together with female gender, predict a higher dose requirement for Gla-100 to achieve target glycemic control at week 24. Longer duration of diabetes and older age predict a lower Gla-100 dose to achieve target glycemic control at week 24.

Multivariate regression analysis in the subpopulation of patients from China demonstrated that BMI, FPG, age, and number of OADs at baseline were significantly associated with the dose of Gla-100 at week 24 (Fig. 1 and Table S2). As with the Asian population, increases in BMI by 1 kg/m^2^ and FPG by 1 mmol/L at baseline were associated with an increase in the dose of Gla-100 at week 24 by 1.25 U (*P* < 0.0001) and 1.43 U (*P* < 0.0001), respectively. Age and number of OADs, however, were negatively associated with the dose of Gla-100 at week 24. An increase in age by 1 year and treatment with one more type of OAD at baseline were associated with a decrease in the dose of Gla-100 at week 24 by 0.31 U (*P* < 0.0001) and 4.78 U (*P* = 0.0032), respectively.

### Secondary Outcomes

**Fig. 3:**
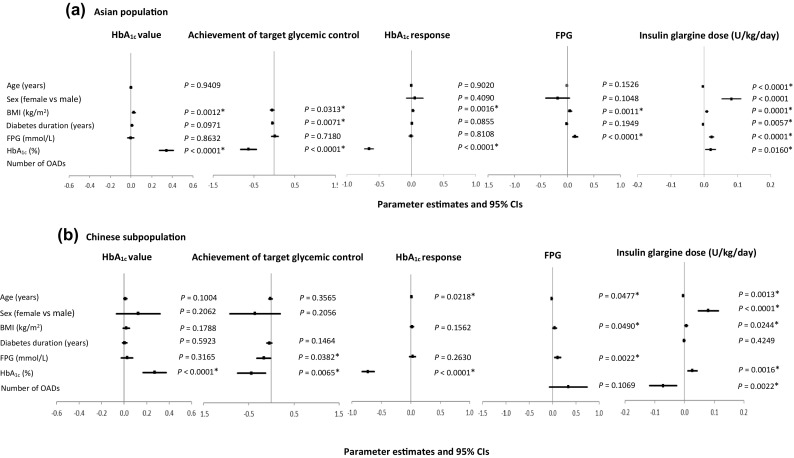
Parameter estimates and 95% confidence intervals for baseline predictors of secondary outcomes at week 24 following multivariate analyses. Results are summarized for covariates included in the final model for each outcome/population. Baseline covariates not included in the multivariate analyses for the different outcomes in both populations have been left blank. *BMI* body mass index, *CI* confidence interval, *FPG* fasting plasma glucose, *HbA*_*1c*_ glycated hemoglobin, *OADs* oral antidiabetic drugs. *Statistically significant

Multivariate analyses were also conducted to determine the impact of patient baseline covariates on treatment response and the dose of Gla-100 in units per kilogram per day (Fig. 3a for the Asian population and Fig. 3b for the Chinese subpopulation).

Multivariate analysis identified the following baseline predictors for HbA_1c_ at week 24: BMI (*P* = 0.0012) and HbA_1c_ (*P* < 0.0001) in the Asian population and HbA_1c_ (*P* < 0.0001) in the Chinese subpopulation. Baseline predictors for achievement of target glycemic control at week 24 were BMI (*P* = 0.0313), duration of diabetes (*P* = 0.0071), and HbA_1c_ (*P* < 0.0001) in the Asian population and HbA_1c_ (*P* = 0.0065) and FPG (*P* = 0.0382) in the Chinese subpopulation. Baseline predictors of HbA_1c_ response at week 24 were BMI (*P* = 0.0016) and HbA_1c_ (*P* < 0.0001) in the Asian population and HbA_1c_ (*P* < 0.0001) and age (*P* = 0.0218) in the Chinese subpopulation. Similarly, for FPG value at week 24, baseline predictors were identified as BMI (*P* = 0.0011) and FPG (*P* < 0.0001) in the Asian population and age (*P* = 0.0477), BMI (*P* = 0.0490), and FPG (*P* = 0.0022) in the Chinese subpopulation.

In terms of the dose of Gla-100 in units per kilogram per day at week 24, identified baseline predictors were HbA_1c_ (*P* = 0.0160), age (*P* < 0.0001), sex (*P* < 0.0001), BMI (*P* = 0.0001), FPG (*P* < 0.0001), and duration of diabetes (*P* = 0.0057) in the Asian population. Identified predictors in the Chinese subpopulation were age (*P* = 0.0013), sex (*P* < 0.0001), BMI (*P* = 0.0244), HbA_1c_ (*P* = 0.0016), and number of OADs (*P* = 0.0022).

## Discussion

Using data from seven published individual TTT RCTs [1, 21–27], this pooled analysis aimed to determine the impact of patient baseline covariates on the dose of Gla-100 and its treatment response at week 24 in an overall population of Asian patients with T2DM and in a subpopulation of these patients from China. Identification of the predictors of dose and treatment response in the Chinese T2DM subpopulation was important to lay the foundation for future research specific to these individuals and to help guide local clinical practice. Results from the Chinese subpopulation were not intended as a comparison with the results obtained in the overall Asian population. For the purpose of this discussion, common baseline predictors for Asian patients and those from China have been elaborated on. Where deemed to be clinically relevant, predictors in the overall Asian population have also been highlighted. However, differences in observed results between the main population and subpopulation may have been affected by several factors, including the number of patients in each group. Further research and clinical validation of the results are therefore required to understand these differences and so are not discussed here.

The dose of Gla-100 at week 24 was negatively correlated with age and positively correlated with BMI and FPG in both the overall Asian population and the Chinese subpopulation. A post hoc analysis of the insulin glargine (Lantus^®^) vs. insulin detemir (Levemir^®^) Treat-To-Target (L2T3) study, one of the RCTs included in this analysis, also demonstrated a similar relationship between final basal insulin dose (glargine or detemir) and the baseline characteristics of age, BMI, and FPG in patients with T2DM [28]. In the post hoc analysis, two stepwise regression analyses were performed, the first of which demonstrated that statistically significant predictors of a high final basal insulin dose, amongst others, were high FPG and younger age [28]. The second regression analysis, in which only physical characteristics that could be assessed “at the bedside” were included, demonstrated that high BMI, high FPG, and younger age were predictors of a high final basal insulin dose [28]. The study acknowledged that rough estimations of the final basal insulin dose can be made when taking into account a patient’s BMI and age [28].

Similar to the primary outcome, the dose of Gla-100 expressed in units per kilogram per day at week 24 was also shown to be negatively correlated with age and positively correlated with BMI and FPG in the Asian population. Post hoc analysis of the L2T3 study also reported a positive correlation between BMI and the final basal insulin dose expressed in terms of weight; however, data supporting this outcome were not shown [28].

These results suggest that a patient with higher FPG at baseline requires a higher dose of Gla-100 to reduce their FPG level, which is in accordance with clinical expectations. The relationship between BMI and dose of Gla-100 has a physiological basis. Higher BMI has been shown to be associated with decreased insulin sensitivity [29, 30], and a higher dose may be required in patients with higher BMI in order to stimulate insulin absorption and decrease glycemic levels. Indeed, titration based on a patient’s weight (an important factor in determining BMI) is thought to have a stronger physiological basis than glucose level or dose-based regimens, especially given the relationship between body weight and insulin sensitivity [31]. As mentioned above, the post hoc analysis of the L2T3 study also demonstrated a negative correlation between age and the final dose of basal insulin; however, the authors of the study acknowledged that there are no straightforward explanations for this finding [28]. The authors of the current study hypothesize that younger patients, who are in need of stricter glycemic control [4, 5, 32] and who have been shown to be at a lower risk of hypoglycemia [33–35], are likely to have had their doses of Gla-100 titrated to a greater extent than older patients, leading to the observed relationship between the dose of Gla-100 at week 24 and age.

The above analyses indicate that a higher dose of basal insulin is likely to be required in younger patients or patients with higher baseline BMI or FPG, and physicians must therefore titrate the insulin doses of these patients to an adequate level to achieve glycemic targets. The opposite must also be taken into consideration in older patients or patients with lower baseline BMI or FPG, for whom lower doses of basal insulin may be required to achieve glycemic targets. In these patients, a more cautious approach to the titration of basal insulin is warranted.

The nomogram developed in this study can help clinicians to determine the dose of Gla-100 likely to be required by a patient to achieve target glycemic control at week 24, thereby providing guidance for the appropriate titration of Gla-100 according to a TTT algorithm. Further details on interpretation of the nomogram are included in Fig. 2. The nomogram developed in this study is a representation of the results of the multivariate analysis of the primary outcome as a whole and includes all the patient baseline covariates included in the analysis, regardless of whether they were significant or not. Therefore, although not significant, baseline HbA_1c_, duration of diabetes, and gender have also been included in the nomogram. With regard to baseline HbA_1c_, post hoc analysis of the L2T3 study demonstrated similar results, with multivariate analyses demonstrating that the baseline level of HbA_1c_ was not a significant determinant of the final basal insulin dose [28]. It should be noted that the dose of Gla-100 predicted by the nomogram developed in this study is only an estimation of the required dose to achieve target glycemic control at week 24 and does not enable calculation of the initial therapeutic dose of Gla-100. In general, Asian country-specific guidelines recommend a dose of 0.1–0.3 U/kg/day for the initiation of basal insulin [6, 36–38].

A formula for determining the total optimal daily dose of Gla-100 at 24 weeks, based on both the optimal starting and incremental doses, has been previously developed using data from a 24-week observational study of Japanese patients with T2DM [39]. The objective and parameters included in the formula differ, however, from those of the nomogram developed in the present analysis; furthermore, the formula was derived from observational data from a single study wherein physicians were free to titrate according to their clinical practice [39]. The nomogram in the current study was developed using data from seven RCTs, each with its own starting doses and TTT algorithms based on FPG [1, 21–27]. The patient populations used in both analyses also differ; the aforementioned formula was derived from a population that achieved target HbA_1c_ [39], whereas the nomogram in the current study was constructed using data from the FAS populations of the individual TTT RCTs. Hence, direct comparisons between the formula and the nomogram developed in the current study cannot be made.

The multivariate analyses demonstrated a statistically significant positive correlation between HbA_1c_ value at baseline and HbA_1c_ value at week 24 in both the Asian population and the Chinese subpopulation. In a study by Fujita et al. [40], multiple linear regression analysis was conducted to determine the characteristics influencing the effectiveness of Gla-100 treatment in insulin-naïve T2DM Japanese patients with suboptimal glycemic control on OADs. HbA_1c_ at baseline was shown to be a statistically significant predictor of HbA_1c_ at week 24 (*P* = 0.006), with an increase at baseline predicting a higher endpoint HbA_1c_ value [40].

In the present study, multivariate analyses also demonstrated a negative association between achieving target glycemic control (HbA_1c_ < 7%) at week 24 and baseline HbA_1c_ in the Asian population and Chinese subpopulation. The negative association between achieving target glycemic control and baseline HbA_1c_ has been reported in several studies aimed at identifying the characteristics associated with glycemic response to newly initiated insulin therapy in both Asian and Western populations with T2DM [41–43]. In particular, a subject-level meta-analysis of 12 RCTs that used Gla-100 in a TTT titration regimen in patients with T2DM, including some of the RCTs involved in the current pooled analysis, demonstrated that baseline HbA_1c_ was negatively associated with achieving HbA_1c_ ≤ 7.0% [43]. In an observational study of newly initiated insulin therapy in patients with T2DM by Nichols et al. [42], multivariate analyses demonstrated that HbA_1c_ prior to insulin initiation was the dominant factor in predicting treatment goal attainment (HbA_1c_ < 7%), and that a 1% increase in HbA_1c_ prior to insulin initiation reduced the probability of attaining target glycemic control by 26% [42]. In the current analysis, a 1% increase in HbA_1c_ prior to initiation of Gla-100 reduced the probability of attaining target glycemic control by 63.1% in the Asian population and by 43.7% in the Chinese population.

In addition to baseline HbA_1c_, duration of diabetes was also negatively associated with achieving target glycemic control at week 24 in Asian patients. The aforementioned studies by Fujita et al. [40] and Nichols et al. [42] have also reported that a longer duration of diabetes is associated with a lower likelihood of achieving target glycemic control.

Validating the above, multivariate analyses conducted in this study demonstrated a negative correlation between HbA_1c_ response (reduction from baseline to week 24) and baseline HbA_1c_ in both the Asian population and the Chinese subpopulation. Predictors of change in HbA_1c_ were also investigated using data from the large, 24-week, observational A_1_chieve study, which involved patients with T2DM initiating insulin therapy. In both predictor and explanatory analyses, HbA_1c_ level at baseline was negatively associated with change in HbA_1c_ from baseline to the endpoint [44].

This above evidence indicates that baseline HbA_1c_ is an important factor in determining the level of glycemic control attained. In the study by Nichols et al. [42], HbA_1c_ prior to starting insulin therapy accounted for 95% of the discriminatory ability to predict the probability of attaining target glycemic control and 96% of the explainable variance in HbA_1c_ change. These observations are expected, since a patient with HbA_1c_ closer to 7% should achieve target glycemic control more easily following treatment initiation with insulin. This, however, does not eliminate the fact that in several studies, patients with T2DM who achieved target glycemic control also had a greater reduction in HbA_1c_ after initiating insulin therapy, despite a lower mean HbA_1c_ at baseline [41, 42].

Baseline predictors for FPG value at week 24 were also investigated in our study. To the best of our knowledge, other studies demonstrating similar results have not been conducted, and hence these novel findings require further investigation. Common baseline predictors in the Asian population and Chinese subpopulation were BMI and FPG, both being positively associated with FPG at week 24.

There are several limitations to this study. No methods were used to assess risk of bias within the individual studies or across the included studies. Additionally, selection of the patient baseline covariates included in the analyses was limited by the available data in the individual RCTs. This pooled analysis, however, provides results from a larger group of Asian and Chinese patients compared with each of the individual seven studies. Additionally, this study performed separate analyses for patients from Asia and for the subpopulation of patients from China, in order to explore the relationship between patient characteristics and outcomes in each of the populations. Understanding the reasons behind differences between the populations’ results requires further research involving larger patient populations and validation of these results. The nomogram developed in this study requires further independent clinical validation. Future studies could also look to develop and assess nomograms based on a wider range of patient characteristics, such as the presence of diabetes complications or comorbidities. Additional research involving a larger pool of baseline covariates, including threshold homeostasis model assessment (HOMA) of insulin resistance levels, HOMA of β-cell function levels, and C-peptide may provide more insight into the impact of insulin resistance and secretion capacity on the dose of Gla-100 and treatment response. Investigating different patient profiles could also explain and establish more in-depth and clinically relevant correlations, such as those associated with the risk of occurrence of hypoglycemia.

## Conclusions

This pooled analysis identified the main baseline predictors of important therapeutic parameters, such as the dose of Gla-100 and treatment response at week 24 in an overall population of Asian patients with T2DM and a subpopulation of these patients from China. In both populations, results indicated that a higher dose of Gla-100 is likely to be required in younger patients, or in patients with higher baseline BMI or FPG, and that physicians must titrate the doses of basal insulin for these patients to an appropriate level in order for them to achieve glycemic targets. Aiding appropriate titration, the nomogram developed in this study enables calculation of the dose of Gla-100 likely to be required by Asian patients with T2DM to achieve HbA_1c_ < 7% at week 24. In both the study populations, higher baseline HbA_1c_ was associated with lower reduction in HbA_1c_ from baseline, higher HbA_1c_ at week 24, and a lower chance of achieving target glycemic control at week 24; this indicates that baseline HbA_1c_ is an important factor in determining the level of glycemic control attained following treatment with Gla-100. The achievement of target glycemic control at week 24 was also negatively associated with duration of diabetes in the Asian population, demonstrating the importance of timely initiation of basal insulin.

## Electronic supplementary material

Below is the link to the electronic supplementary material.

Supplementary material 1 (PDF 102 kb)
