# Supplementary material for: Insulin Glargine Combined with Oral Antidiabetic Drugs for Asians with Type 2 Diabetes Mellitus: A Pooled Analysis to Identify Predictors of Dose and Treatment Response
Source: Diabetes Ther. 2018 Mar 9;9(2):771–87. doi: 10.1007/s13300-018-0381-9 (PMC6104270; doi:10.1007/s13300-018-0381-9)
Supplement: Supplementary file 1 — Supplementary material 1 (PDF 102 kb) [file 13300_2018_381_MOESM1_ESM.pdf]

Table S1. Patient disposition

Category	N (%)
**Number of included subjects**	724
**Number of included studies**	7
**Number of subjects in each study (Asian population)**	
ATLAS	403 (55.7)
GALAPAGOS	198 (27.3)
0579	73 (10.1)
4001	14 (1.9)
3502	13 (1.8)
4041	12 (1.7)
4002	11 (1.5)
**Number of subjects in each study (Chinese population)**	
ATLAS	150 (60.2)
GALAPAGOS	99 (39.8)

Table S2. Parameter estimates and 95% confidence intervals for baseline predictors of Gla-100 dose at Week 24 following multivariate regression analyses

Baseline covariate*	All patients (N = 724)		Chinese patients (n = 249)	
	Estimate (95% CI)	*P* value	Estimate (95% CI)	*P* value
Age, years	–0.26 (–0.39, –0.13)	0.0001	–0.31 (–0.45, –0.16)	<0.0001
Sex, female vs male	1.55 (–0.51, 3.62)	0.1406	1.93 (–0.45, 4.31)	0.1108
BMI, kg/m^2	1.44 (1.18, 1.70)	<0.0001	1.25 (0.90, 1.61)	<0.0001
Duration of diabetes, years	–0.23 (–0.41, –0.05)	0.0135		
FPG, mmol/L	1.62 (1.12, 2.11)	<0.0001	1.43 (0.88, 1.98)	<0.0001
HbA_{1c}, %	1.07 (–0.05, 2.18)	0.0602		
Number of OADs			–4.78 (–7.94, –1.61)	0.0032

BMI, body mass index; CI, confidence interval; FPG, fasting plasma glucose; Gla-100, insulin glargine 100 U/mL; HbA_{1c}, glycated hemoglobin; OADs, oral antidiabetic drugs.

*Results are summarized for covariates included in the final model for each population. For the overall Asian population, the number of OADs prescribed at baseline did not satisfy the forward model entry selection criterion (from univariate to multivariate analysis) and hence was not included in the multivariate analysis. Similarly, for the Chinese patients, duration of diabetes and HbA_{1c} at baseline both failed to satisfy the entry criterion from univariate to multivariate analyses. Baseline covariates not included in the multivariate analyses for both populations have been left blank.
